# Effect of interleukin-1 antagonist on growth of children with colchicine resistant or intolerant FMF

**DOI:** 10.1186/s12969-022-00784-6

**Published:** 2023-01-09

**Authors:** Shiran Pinchevski-Kadir, Maya Gerstein, Oren Pleniceanu, Yonatan Yacobi, Asaf Vivante, Ortal Erez Granat, Shiri Spielman, Rotem Semo Oz, Irit Tirosh

**Affiliations:** 1 Department of Pediatrics B, The Edmond and Lily Safra Childrens Hospital, Sheba Medical Center, Tel Hashomer, Israel; 2 Pediatric Rheumatology unit, The Edmond and Lily Safra Childrens Hospital, Sheba Medical Center, Tel Hashomer, Israel; 3 Sackler Faculty of Medicine, Tel-Aviv University, Tel-Aviv, Israel; 4 Department of Internal Medicine D, Sheba Medical Center, Tel Hashomer, Israel; 5 Department of Pediatrics A, The Edmond and Lily Safra Childrens Hospital, Sheba Medical Center, Tel Hashomer, Israel

**Keywords:** FMF, Anti-IL-1, Growth

## Abstract

**Introduction:**

Familial Mediterranean Fever (FMF) is the most common monogentic autoinflammatory disease. FMF results from mutations in *MEFV*, which lead to a pro-inflammatory state and increased production of Interleukin 1 beta subunit (IL-1b) by myeloid cells. Despite the overall positive results obtained with anti-IL-1 agents in FMF patients, little is known about the long-term growth impact of these drugs in the pediatric population.

**Objectives:**

To assess the long-term body weight and height trajectories in children with FMF treated with anti-IL-1 agents.

**Methods:**

We conducted a retrospective analysis of 646 pediatric FMF patients followed in our center, of whom 22 were treated with either anakinra (36.3%) and/or canakinumab (90.9%). Patients were assessed for demographic, clinical and genetic characteristics and were followed for a mean of 3.05 ± 1.75 years. Data of height and weight percentiles were recorded before and after treatment.

**Results:**

The most common indication for IL-1 blockers treatment was colchicine resistance (66.6%). Ninety percent of those patients had a moderate or severe disease according to the Pras score and had higher proportion of M694V homozygosity compared with patients who did not require anti IL-1 agents (95.2% vs. 30.5%, *p* < 0.001). Overall, anakinra and canakinumab resulted in a complete response in 80% of patients and exhibited low rates of adverse effects. We found a significant increase in height and body weight percentiles following treatment (19.6 ± 16% vs. 30.8 ± 23%, *p* = 0.007, and 29.5 ± 30% vs. 39.1 ± 36%, *p* = 0.043, respectively).

**Conclusion:**

Treatment with anti-IL-1 agents in children with FMF is effective and safe and may potentiate long-term growth.

## Introduction

Familial Mediterranean Fever (FMF) represents the most common form of monogenic autoinflammatory disease [1]. It is characterized by recurrent, self-limited, inflammatory attacks, lasting 1–3 days. FMF results from gain of function mutations in the *MEFV* gene located on chromosome 16 which encodes pyrin, a 781-amino acid long protein expressed primarily in cells of the innate immune system. There, it serves as a negative regulator of a cytosolic cellular multiprotein complex termed the inflammasome, which mediates the activation of various inflammatory pathways in response to deleterious cellular insults (e.g., infection) and drives the maturation of the IL-1b and IL-18. Accordingly, dysregulated inflammasome activity results in a pro-inflammatory state and has been linked to autoinflammatory diseases [2]. In FMF, the added pyrin activity leads to an unrestricted inflammasome activation, thereby resulting in a hyperinflammatory state [2, 3]. Among the different mutations described in *MEFV*, the most common is M694V, which has been shown to present in 20–65% of patients. Importantly, M694V homozygotes suffer from a more severe form of the disease, including frequent episodes and the need for higher colchicine doses to prevent attacks, as compared to patients with other mutations [4]. Approximately 10–20% of individuals who meet diagnostic criteria for FMF have no identified mutations in the *MEFV* gene [5]. Colchicine, which still represents the mainstay of FMF treatment, has been shown to prevent FMF attacks in 60–65% of patients and to result in a partial remission in an additional 30–35% of patients [6]. Moreover, colchicine is capable of eliminating the long-term risk of secondary amyloidosis, which is the most significant complication in FMF. An additional complication of FMF in the pediatric population is the negative impact on linear growth [7], similar to observations in other chronic diseases in children [8, 9]. Indeed, it has been shown that successful treatment with colchicine has a positive effect on both height and weight parameters in children with FMF [10–12]. These studies suggest that colchicine improves growth by suppressing disease activity and inflammation. Yet, up to 10% of FMF patients do not respond to colchicine treatment, even under maximal doses and full compliance [13]. In recent years, a growing understanding of the molecular basis behind FMF has resulted in a new generation of drugs, directed against IL-1. These include anakinra, which is a recombinant form of the naturally occurring IL-1 receptor antagonist [14], and canakinumab, a high affinity, fully human monoclonal anti-IL-1b antibody [15]. Both drugs are currently prescribed to colchicine-resistant or -intolerant FMF patients, although colchicine is still used in these patients to prevent amyloidosis, as the two biological drugs have not been consistently shown to have this effect. Both anakinra and canakinumab have a relatively favorable safety profile, with the most common side effects being injection site reactions and infections (typically of the upper respiratory tract) [16, 17]. Despite the growing use in anti-IL1 drugs in FMF among children, data is still lacking in regard to long-term follow up. In addition, the information regarding the impact of these novel drugs on linear growth in children with FMF is minimal [18]. Herein, we carried out a single center retrospective cohort study involving 22 pediatric FMF patients treated with anti-IL1 agents to evaluate their long-term growth response to treatment in addition to assessment the overall efficacy and safety of these agents.

## Methods

### Study design and population

We carried out a historical cohort study by collecting and analyzing data of 646 pediatric FMF patients treated at Sheba Medical Center in Israel between the years 2006–2022, including 22 patients who were treated with anti-IL-1 agents, anakinra and/or canakinumab Patients were started Anakinra only when Canakinumab was not approved by their health insurance as their first biologic agent. All patients were diagnosed according to Tel-Hashomer criteria [19]. Demographic and clinical data including age, sex, age at first symptom, age at diagnosis, symptoms, reason for commencing biological treatment, type and time of onset of biological treatment and clinical response to biological treatment, were collected retrospectively from medical charts of patients treated with anti IL1 agents. Height and weight measurements before and after biological treatment were obtained only for patients who started the biologic treatment under the age of 16 years. Height and weight percentiles were calculated according to the Centers for Disease Control and prevention (CDC) growth charts. In addition, we obtained the genetic information regarding the presence and type of mutation in *MEFV*, for those who had undergone genetic testing. Patients were followed until the end of the study (2022) or until treatment cessation (which was either due to treatment failure and change in treatment, or non-compliance). We also extracted any documented side effects, based on description of symptoms, physical examination, and/or laboratory tests.

### Response to treatment

We classified the response to treatment as complete response (complete absence of attacks with normal levels of inflammation markers), partial response (a decrement in severity, frequency, and duration of attacks but with no complete response) and no response.

### Disease severity score and follow-up

Disease severity was calculated according to Pras severity criteria [20]. Pras Score reflects the perspective of the disease course and includes the maximal colchicine dose used, maximal frequency of attacks and an overview of the entire clinical manifestations of the disease throughout the follow-up period and therefore was calculated at the patients’ last appointments. Disease severity is defined as follow: mild disease 2–5 points, moderate disease 6–10 points, severe disease > 10 points.

### Statistical analysis

Results are presented as mean ± standard deviation or as proportion (%) as indicated. Differences between the groups in discrete variables were evaluated by Chi-square test. Comparison of genetic variants between groups was conducted using the Fisher Exact test. Paired Student t test was used to compare pre and post treatment differences in continuous variables (height and weight percentiles). Normality of body weight and height was verified using D’Agostino and Pearson test. A two-tailed *p*-value < .05 was considered significant. All statistical analyses were calculated using prism graphpad v.9.

### Ethics approvals

This study was approved by the Institutional Review Board of Sheba Medical Centre.

## Results

### Study population

We retrospectively collected data from a cohort of 646 pediatric FMF patients treated at our Center between the years 2006–2022. Among these, 22 patients (3.4%) required treatment with an anti-IL1 agent. Over the years of follow-up, 20 (90.9%) of whom were treated with canakinumab and 8 (36%) were treated with anakinra (Table 1), as few patients were sequentially exposed to both medications. The 22 patients (63.6% females) were diagnosed at a mean age of 3.68 ± 2.04 years, and anti-IL1 treatment was initiated at a mean age of 12.85 ± 4.2 years. Mean follow-up was 3.05 ± 1.75 years (Minimum 1 year, Maximum 7.25 years) (Table 1). According to the Pras severity score, 36.3% had severe disease, 54.5% had moderate disease, and 9% were classified with mild disease. Colchicine resistance was the most common cause (66.6%) for initiating treatment with anti-IL-1 drug, followed by colchicine intolerance (52.3%).

**Table 1.**
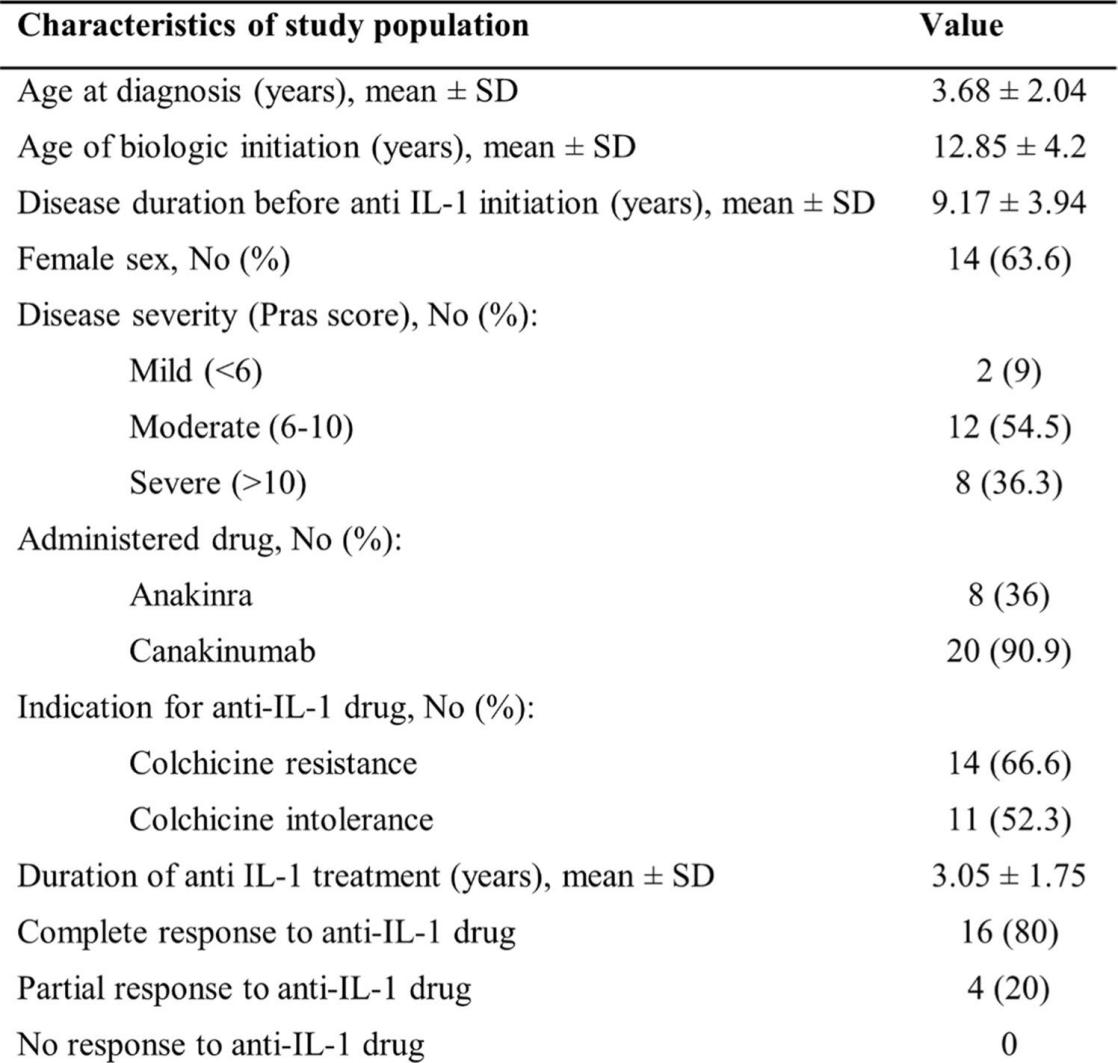
Summary of demographic and clinical characteristics of 22 pediatric patients treated with anti-IL-1 agents

### Genotype of patients treated with anti-IL1 agents

Of the 646 pediatric FMF patients in our cohort, 517 patients had available information regarding their *MEFV* genotype. This included 173 (33.4%) patients who were homozygous, 145 (28%) who were compound heterozygous, 172 (33.2%) who were heterozygous and 27 (5.2%) individuals in whom no mutation was identified. Mutation analysis was available for 21 out of the 22 patients who were treated with anti-IL-1 agents. Thus, we compared the mutation’s distribution between these 21 patients to our genetically identified FMF cohort. As indicated in Table 2, there was a significantly higher rate of M694V homozygosity among anti-IL-1 treated patients, compared to colchicine-treated patients (95.2% vs. 30.5%, *p* < 0.0001). In fact, 12.6% of the patients harboring M694V homozygous mutations were eventually treated with anti-IL-1 agents. One additional patient in the anti-IL-1 group was a compound heterozygote for M694V/V726A.

**Table 2.**
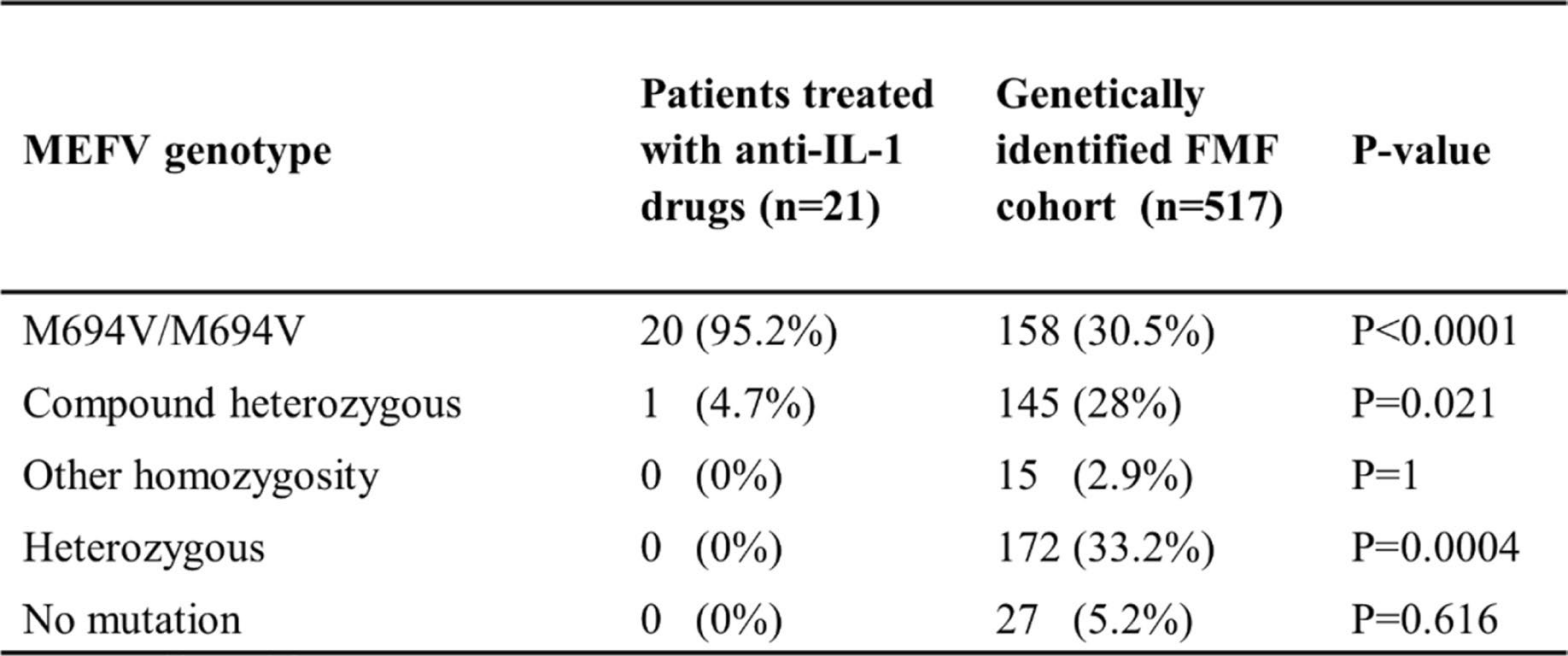
Genotype of the study population.

### Treatment outcomes in patients treated with anti-IL1 agents

**Fig. 1:**
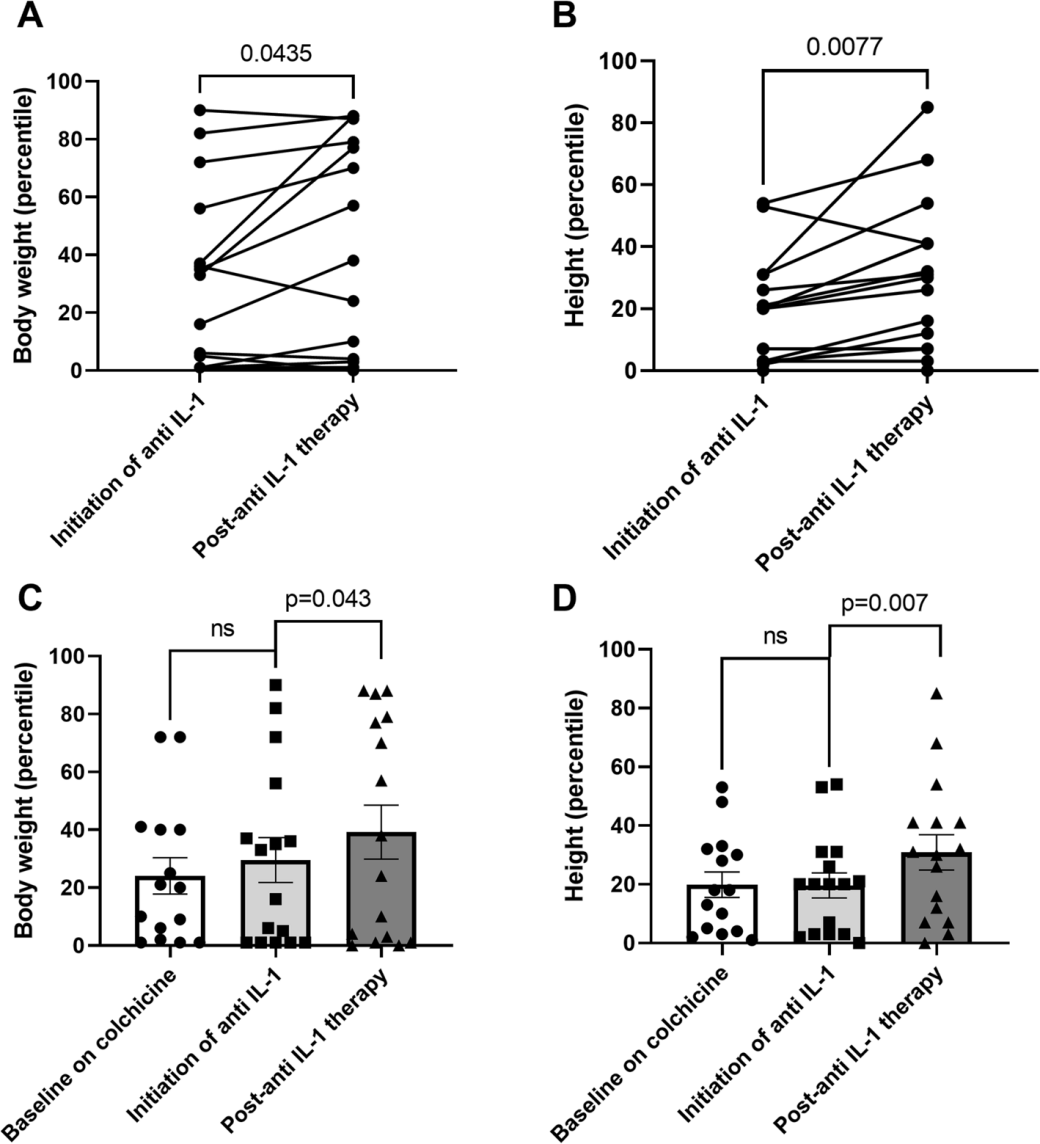
A significant increase in body weight (A) and height (B) percentiles following treatment initiation. Baseline on colchicine measurements of body weight (C) and height (D) 1-year prior to anti IL-1 treatment demonstrate non-significant increase compared to significant increase following treatment

Overall, 80% of patients treated with these agents demonstrated complete response, with an additional 20% showing partial response with none had no response (Table 1), two patients were lost to follow up and we do not have their response to treatment data. Next, we examined the effect of anti-IL-1 drugs on changes in body weight and height percentiles. Given the minimal change in linear growth at late stages of puberty, we included for this analysis only 16 patients who initiated biologic treatment before the age of 16 years. We observed a significant increase in both body weight **(**Fig. 1A**)** and height **(**Fig. 1B**)** percentiles following treatment initiation (29.5 ± 30% vs. 39.1 ± 36%, *p* = 0.043, and 19.6 ± 16% vs. 30.8 ± 23%, *p* = 0.007, respectively), with 56 and 75% of treated children demonstrating an increase in body weight and height percentiles at the end of the follow-up, respectively. We next conducted an additional analysis comparing growth percentiles in the year before initiation of biologic treatment, while all patients were actively treated with colchicine, with baseline growth parameters. As demonstrated in Fig. 1C and Fig. 1D, the significant increase in body weight and height percentiles, respectively, was recorded only following initiation of anti IL-1 therapy.

Lastly, we assessed the safety of anti-IL1 treatments in our cohort. Overall, during 771 patient months of follow-up, one patient reported a local skin reaction at the injection site, and two patients complained of abdominal pain. Notably, no additional side effects were noted.

## Discussion

Anti-IL-1 agents are highly effective and safe in patients with colchicine-resistant FMF [17, 21, 22]. However, the efficacy and safety profile of these drugs in the treatment of pediatric FMF is largely limited to a relatively short duration of follow-up. To the best of our knowledge, this study provides efficacy and safety data from the longest follow-up to date, with reassuring evidence that this group of drugs is extremely well tolerated and results in excellent clinical outcomes, with 80% achieving complete treatment response.

The main finding of our study is the significant increase in both height and body weight percentiles among FMF patients treated with anti-IL-1 drugs. Our results corroborate with historical reports demonstrating a positive effect of colchicine on growth and development in pediatric FMF patients [10–12]. Of interest, one study demonstrated that an earlier initiation of colchicine resulted in a larger positive effect on linear growth [12]. This finding could be explained by an earlier alleviation of the deleterious effects of chronic diseases in general and inflammatory disorders in particular, on linear growth [23, 24].

Two previous studies reported the influence of canakinumab on linear growth in autoinflammatory conditions in children. In the study of Balci et al. of 11 children with FMF, despite a median follow-up of only 1.5 years, canakinumab treatment had a positive effect on both height and body weight Z-scores [18]. Yücel et al., however, have failed to demonstrate a positive effect of canakinumab on linear growth and reported an improvement in body weight Z-scores only [17]. This lack of improvement in height percentiles in this study was attributed by the authors for late initiation of treatment and a relatively short duration of follow-up. Taken together with our results, one may argue in favor of early initiation of anti-IL-1 agents in colchicine resistant FMF patients not only for better control of inflammatory attacks but also to maximize their linear growth potential.

There are different reports in the literature regarding the percentage of patients who had complete response to anti IL-1 agents. Complete remission following canakinumab treatment was reported by several groups, such as Brik et al. who reported 42.9% with complete response [25], De Benedetti et al. [26], and Kisla Ekinci et al. [27] reporting both of ~ 70% with complete response, with some reporting a complete response in up to 100% of cases [22, 28]. This difference could be related to variation in treatment regimen between studies and distribution of *MEFV* mutations in the various cohorts.

Interestingly, in this study we report a very low rate of side effects, despite a mean follow-up of more than 3 years. One patient reported an injection site skin reaction, and two additional patients complained of abdominal pain. These results are in-line with published data regarding canakinumab safety [17, 22, 27]. It is worth noting that in a randomized, placebo-controlled trial of canakinumab in autoinflammatory diseases in children, a somewhat higher rate of adverse events was recorded, including infections (23.8%), injection site reactions, (6.0%), headache (3.9%) and abdominal pain (3.6%) [26]. In particular, infections have previously been reported in some studies to occur in patients treated with anti-IL-1 drugs, including major bacterial infections (e.g. pneumonia [20, 29]). Nevertheless, in our study, none of the patients reported of significant infections during their routine follow-up visits. In addition, none of our patients discontinued the treatment due to side effects, similar to what was described in a systematic review of 19 studies of canakinumab [21].

As for the genetic profile of pediatric FMF children, our findings are consistent with the more severe phenotype described in patients who are homozygous for the M694V mutation [30]. We found that 95% of FMF patients who required the use of anti-IL-1 agents were homozygous to the M694V mutation, as opposed to only 30.5% in the entire genetic cohort at our Center. In fact, 12.6% of the patients harboring the M694V homozygous mutation were eventually treated with anti-IL-1. Thus, patients with severe disease course who are not adequately controlled by colchicine, especially those who are homozygotes for the M694V mutation, should be considered for an early intervention with anti-IL1 agents, in order to gain maximal benefits of this treatment regimen on linear growth.

This study has several limitations that require consideration. First, the retrospective design does not allow us to infer on causality. Second, the relatively small number of patients limits our ability to address the age and sex specific effects of anti-IL-1 drugs on growth. Yet, our comprehensive database with detailed clinical parameters of over 600 FMF patients treated at our institution and a mean follow-up of over 3 years provide us with a unique opportunity to conclude regarding the long-term anthropometric effects of anti-IL-1 agents in children with FMF.

## Conclusions

Our study shows that anti -IL-1 agents represent an effective and safe treatment and may improve growth in children with colchicine-resistant FMF. Therefore, we suggest considering early treatment when needed, especially for those who harbor the M694V homozygous mutation.

## Data Availability

The datasets used in the current study are available from the corresponding author on reasonable request.

## Abbreviations

FMF: familial Mediterranean fever
IL-1b: Interleukin 1 beta subunit
WHO: world health organization.
